# Proteophosophoglycans Regurgitated by *Leishmania*-Infected Sand Flies Target the L-Arginine Metabolism of Host Macrophages to Promote Parasite Survival

**DOI:** 10.1371/journal.ppat.1000555

**Published:** 2009-08-21

**Authors:** Matthew Rogers, Pascale Kropf, Beak-San Choi, Rod Dillon, Maria Podinovskaia, Paul Bates, Ingrid Müller

**Affiliations:** 1 Department of Immunology, Imperial College of Science, Technology and Medicine, London, United Kingdom; 2 Vector Group, Liverpool School of Tropical Medicine, Liverpool, United Kingdom; 3 Department of Infectious and Tropical Diseases, London School of Hygiene and Tropical Medicine, London, United Kingdom; 4 Department of Molecular and Biochemical Parasitology, Liverpool School of Tropical Medicine, Liverpool, United Kingdom; National Institute of Allergy and Infectious Diseases, United States of America

## Abstract

All natural *Leishmania* infections start in the skin; however, little is known of the contribution made by the sand fly vector to the earliest events in mammalian infection, especially in inflamed skin that can rapidly kill invading parasites. During transmission sand flies regurgitate a proteophosphoglycan gel synthesized by the parasites inside the fly midgut, termed promastigote secretory gel (PSG). Regurgitated PSG can exacerbate cutaneous leishmaniasis. Here, we show that the amount of *Leishmania mexicana* PSG regurgitated by *Lutzomyia longipalpis* sand flies is proportional to the size of its original midgut infection and the number of parasites transmitted. Furthermore, PSG could exacerbate cutaneous *L. mexicana* infection for a wide range of doses (10–10,000 parasites) and enhance infection by as early as 48 hours in inflamed dermal air pouches. This early exacerbation was attributed to two fundamental properties of PSG: Firstly, PSG powerfully recruited macrophages to the dermal site of infection within 24 hours. Secondly, PSG enhanced alternative activation and arginase activity of host macrophages, thereby increasing L-arginine catabolism and the synthesis of polyamines essential for intracellular parasite growth. The increase in arginase activity promoted the intracellular growth of *L. mexicana* within classically activated macrophages, and inhibition of macrophage arginase completely ablated the early exacerbatory properties of PSG *in vitro* and *in vivo*. Thus, PSG is an essential component of the infectious sand fly bite for the early establishment of *Leishmania* in skin, which should be considered when designing and screening therapies against leishmaniasis.

## Author Summary

Parasites are known to manipulate their arthropod vectors for increased transmission, yet little is known about the manipulator-molecules involved. The protozoan parasite *Leishmania* secrete a proteophosphoglycan-rich gel (termed promastigote secretory gel, PSG) to block the sand fly midgut to force the regurgitation of parasites and gel into the skin. Here we show that the amount of PSG and dose of *Leishmania* transmitted by individual sand flies strongly correlate with the size of the original midgut infection. Regurgitated PSG exacerbated both low and high dose mouse infections, resulting from the gel's ability to augment the recruitment of its principal host cell, the macrophage, to the site of injury and induce the upregulation of macrophage arginase activity. The infecting parasites take advantage of the increased arginase-mediated L-arginine catabolism and the increased pool of polyamines available within these macrophages for their early nutrition and growth. This resulted in enhanced survival and growth of *Leishmania* in macrophages. Since arginase plays a crucial role in orchestrating wound repair in skin, it would appear that through the generation of PSG, *Leishmania* has evolved to exploit the wound response to the bite of the sand fly for its early survival.

## Introduction

Leishmaniasis is a disfiguring and potentially fatal parasitic infection that affect some 350 million people worldwide [1]. *Leishmania* are obligate intracellular protozoan parasites of macrophages that are transmitted between hosts by the bite of female phlebotomine sand flies and are delivered to a host as mammal-infective metacyclic promastigotes along with the saliva of the sand fly and a mucin-rich gel produced by the parasites in the sand fly midgut [2],[3]. These parasites are rapidly phagocytosed by resident and recruited macrophages within which they transform into, and multiply as aflagellate amastigote forms. It is emerging that both the dose and way in which *Leishmania* are delivered to the mammalian host by the sand fly has considerable significance on the outcome of infection [3]–[6].

Recently, *Leishmania major* infected sand flies were found to inoculate a median of 5,000 to 10,000 parasites; with a minor proportion of flies able to deliver much higher doses of up to 100,000 parasites [4]. Most of these parasites are likely to originate from a gel-like blockage which occludes the anterior midgut of the sand fly [3],[7]. The obstruction, common to all *Leishmania*-sand fly combinations studied to date, is comprised of mucin-like proteophosphoglycans secreted by the parasites, that under high concentrations in the limited volume of the sand fly midgut condense to form the promastigote secretory gel (PSG) [8]. Using the permissive and transmissible *Leishmania*-sand fly vector combination of *Leishmania mexicana* and *Lutzomyia longipalpis* we found this blockage forced the sand fly to clear and regurgitate PSG before it can successfully bloodfeed, thereby depositing both PSG and metacyclic promastigotes into the skin [3]. PSG was found to significantly exacerbate parasite growth and chronic cutaneous lesion development in mice [3]. The active component of PSG was found to be its major constituent, filamentous proteophosphoglycan (fPPG), however, the immune mechanisms by which PSG promote *Leishmania* infection remain unknown.

*Leishmania* have developed a number of strategies to prevent elimination by both innate and adaptive immune effector mechanisms [9]. One such mechanism targets the L-arginine metabolism of macrophages [10],[11]. Macrophages can either kill or host intracellular amastigote forms of *Leishmania* depending on the balance of two inducible enzymes, nitric oxide synthase 2 (iNOS) and arginase-1 which share the same substrate, L-arginine. Both arms of the pathway are controlled by the balance of Th1 (e.g. IFNγ, TNFα) and Th2 (e.g. IL-4, IL-10, IL-13, IL-21) cytokines perceived by the macrophage [12]. Th1 cytokines induce the classical activation of macrophages (CAMΦ), upregulate nitric oxide synthase2 (iNOS) which catabolizes L-arginine into the *Leishmania*-toxic metabolite nitric oxide (NO) [13] in a two-step process. Th2 cytokines alternatively activate macrophages (AAMΦ) and induce arginase-1 which hydrolyzes L-arginine into urea and L-ornithine; the latter being the first building block for the synthesis of polyamines. Polyamines are small cationic molecules required for cell growth and homeostasis. In particular, the metabolism of arginine is essential for wound healing and arginase-induced L-arginine catabolism is a principal constituent of the repair phase following tissue damage [14],[15]. Polyamines are also essential for intra-macrophage growth of *Leishmania* [10],[11].

Following deposition into the skin *Leishmania* parasites may face a range of immune environments that can greatly influence the course of infection. For example, it appears that the early cellular response to the sand fly bite can influence the progression of *Leishmania* infection. In particular an important role for neutrophils has recently been confirmed as an important host cell for *Leishmania* during the first hours of infection [16]. Furthermore, a host's previous exposure to sand fly saliva appears to be important to the course of *Leishmania* infection [17]. It is well established that saliva from the sand fly vector can exacerbate *Leishmania* infection in naïve mice [18], however, vaccination by pre-exposure to the bites of uninfected sand flies [19], with whole saliva [17] or with defined salivary proteins [20]–[22] have shown to protect against cutaneous *L. major* infection. In each example the mode of protection correlates strongly with an IFNγ-dominated cellular response at the dermal site of inoculation. Sand fly bites are known to induce strong delayed type hypersensitivity (DTH), characterized by a rapid infiltration of pro-inflammatory cells of a T-helper1 (Th1) phenotype [20]. Therefore, in humans from an endemic focus it is likely that macrophages recruited to the site of infection are likely to encounter a proinflammatory milieu and high levels of Th1 cytokines which can instruct their activation toward a leishmanicidal state [23].

Here we describe how PSG interferes with the leishmanicidal state of macrophages and identify increased expression of host arginase as the mechanism by which it enhances parasite persistence. Crucially, PSG-mediated host cell modulation allows increased proliferation of *Leishmania* parasites inside macrophages under a range of activation states that may prevail in the skin at the time of, or in response to sand fly transmission.

## Materials and Methods

### Ethics statement

All procedures involving animals were approved by the ethical review committee of Imperial College and performed in accordance with United Kingdom Government (Home Office) and EC regulations.

### Chemicals and reagents

All chemicals and reagents were from Sigma-Aldrich, UK unless stated otherwise.

### PSG and sand fly saliva preparation

Colony reared female *Lu. longipalpis* (Jacobina strain) were infected with *L. mexicana* (MNYC/BZ/62/M379) amastigotes freshly isolated from BALB/c rump lesions as previously described [24]. PSG was dissected from 7 days old fly infections in LPS-free PBS and removed of parasites by 6 rounds of centrifugation (3,300 rpm, 5 min) before freezing at −20°C until required. Saliva was also collected in endotoxin-free PBS by piercing individual salivary glands from 5 days old uninfected female *Lu. longipalpis* maintained on 70% sucrose solution *ad libitum* [3]. All preparations of PSG and saliva were sterilized through 0.22 µm pore syringe filters before use.

### Deglycosylation of PSG

PSG was deglycosylated by mild acid hydrolysis as previously described [3]. Briefly, PSG was acidified to pH 2.0 with 1N HCl, heated at 60°C for 1 hour and neutralised using 1N NaOH.

### Parasite generation

Metacyclic promastigotes of wild type or *LPG2^−/−^* [25] *L. mexicana* (MNYC/BZ/62/M379) were enriched in stationary phase cultures grown from lesion amastigotes as described previously [3]. Transformed promastigotes were grown until Log phase in M199 medium (supplemented with 10% v/v heat-inactivated foetal calf serum (hiFCS, Gibco, UK), 1% v/v penicillin-streptomycin (PS), 1xBME vitamins), pH 7.2 and then passaged into Grace's insect culture medium (Invitrogen, supplemented as for M199 medium) pH 5.5 to reach stationary phase at 26°C. This method has been shown to produce a high yield of *L. mexicana* metacyclic promastigotes (typically 85–90%) that are of equal infectivity to those obtained from infected *Lu. longipalpis* sand flies [3]; without the need for biophysical or biochemical selection. Metacyclic promastigotes were washed extensively in PBS before use.

### Infection of mice

Female BALB/c mice were infected by intradermal injection of 10, 100 or 10^4^ *L. mexicana* metacyclic promastigotes into the shaved rump with or without 1 µg *L. mexicana* PSG. Lesion development was monitored by measuring two diameters of the swelling with Vernier callipers and averaging them. At the end of experiments, mice were humanely euthanized, and parasite burdens in the rump lesions determined by direct counting via hemocytometer.

### Bone marrow macrophage culture and infection

Macrophages were differentiated from bone marrow precursor cells in the presence of L929 fibroblast cell culture supernatant as a source of macrophage colony stimulating factor (CSF-1-responsive bone marrow-derived macrophages) as previously described [11],[26]. Briefly, bone marrow was obtained from femurs from 6–8 week old BALB/c or C57BL/6 mice and bone marrow cells were cultured in supplemented DMEM medium (Gibco) (10% v/v hiFCS, 5% v/v horse serum, 1% v/v PS, 2 mM L-glutamine, 5×10^−5^ M β-mercaptoethanol and 15% v/v L929) at 37°C, 10% CO_2_ in a humidified incubator. Following 8 days of differentiation in hydrophobic Teflon bags the bone marrow derived macrophages (BMMΦ) were washed and seeded into 16 well slides or microtitre plates at 5×10^4^ BMMΦ/200 µl (Lab-Tek, Nunc), or in 24 well culture plates at 5×10^5^/ml. *In vitro* generated macrophages were exposed to *Leishmania* at a parasite to macrophage ratio (multiplicity of infection) of 5 *L. mexicana* metacyclic promastigotes to 1 macrophage (MOI 5∶1) unless stated otherwise for 4 hours to achieve an intermediary level infection to assess the impact of various activations or vector-derived components on infection. Macrophages were washed extensively in DMEM to remove external parasites, then activators (classical activation: 100 U/ml IFN-γ and 500 U/ml TNF-α; alternative activation: 20 U/ml IL-4, Preprotech), *L. mexicana* PSG or *Lu. longipalpis* saliva (1 µg for 5×10^5^ MΦ and 0.25 µg for 5×10^4^ MΦ) were added and the cells were cultured for up to a further 72 hours.

The average intracellular parasite load was determined by oil-immersion microscopy of at least 200 Giemsa-stained macrophages per treatment (performed in duplicate), using the formula: (#parasites/#infected cells)×(#infected cells/total#cells)×100. Infections are expressed as the average number of intracellular parasites per 100 infected macrophages.

### Phagosome-lysosome co-localisation and confocal microscopy

The fusion of late endosomes with lysosomes of C57BL/6 BMMΦ after 4 hours of infection with metacyclic promastigotes of red fluorescent *L. mexicana* (dsRed *L. mexicana*, a kind gift of Dr. Aebischer, Edinburgh [27]) was determined by confocal microscopy. After infection/activation the macrophages were fixed with 4% paraformaldehyde in PBS for 30 min at 4°C, permeabilised for 3 min with 0.1% Triton X-100 in PBS at room temperature (RT) and blocked with 1% bovine serum albumin (BSA), 5% horse serum, 0.1% Tween20, 0.05% sodium azide in PBS for 30 min at RT. Early endosomes were labeled by pulse (4 hours)-chase (16 hours) with dextran-FITC prior to infection and activation. Late endosomes were labeled by incubation of cells with anti-RAB7 (1∶200 dilution in blocking buffer) for 30 min at RT followed by fixation, permeabilisation and blocking. After extensive washing with PBS the cells were incubated for a further 30 min with anti-rabbit Cy5-labelled secondary antibodies (1∶100 in blocking buffer) at RT. The cells were washed again and mounted with embedding medium (Microtech labs) and a coverslip, before being examined with a Zeiss confocal fluorescent microscope using a 63× oil immersion objective. Images were acquired and analyzed by Zeiss LSM image browser

### *In vivo* kinetic of cell recruitment into the air pouch

3 ml of sterile air was injected into the backs of shaved BALB/c mice to inflate an air pouch. Into each air pouch 1 µg PSG, 1 µg *Lu. longipalpis* saliva, or 1×10^3^ *L. mexicana* metacyclic promastigotes were injected in a total of 150 µl endotoxin-free PBS using a 27-gauge needle. This technique allows the differential quantification of leukocyte subsets that transmigrate into the air-pouch cavity in response to the various vector-derived products. At 4, 12, 24, 48 or 72 hours post-injection the cells from the air pouch were recovered using a 5 ml ice-cold medium cavity lavage. Following this, the cells were concentrated to 0.5 ml by centrifugation (1800 rpm, 5 min) and live cells counted by diluting in trypan blue dye using a Neubauer improved haemocytometer. The cells were cytoadhered to glass slides using a Shandon cytospin2 (500 rpm, 5 min) and stained with 10% Giemsa solution to determine the proportions of monocytes/macrophages, neutrophils, lymphocytes, basophils and eosinophils. In some experiments the air pouch was infected directly with 1×10^6^ *L. mexicana* metacyclic promastigotes. To inhibit arginase *in vivo* BALB/c mice were treated with 100 µg *N* ^ω^-hydroxy-nor-L-arginine (nor-NOHA, Bachem, Switzerland) in 0.1 ml endotoxin-free PBS [11]. Mice received daily intraperitoneal injections of nor-NOHA starting 3 days before and during infection.

### Promastigote transformation growth assay

5×10^5^ BALB/c BMMΦ or air pouch macrophages infected for 48 hours in 24 well plates with *L. mexicana* metacyclic promastigotes were lysed to release their amastigotes and grown for a further 48 hours in promastigote medium. Macrophages were lysed with sterile 0.008% v/v SDS in simple DMEM medium for 4 minutes at room temperature. The lysis was stopped by the addition of 17% v/v hiFCS in simple DMEM medium and the cells were mechanically ruptured with a cell scraper. Released amastigotes were concentrated on the bottom of the wells by centrifuging the plate at 3100 rpm for 10 minutes, washed in PBS and resuspended in 200 µl M199 promastigote medium (M199 medium supplemented with 1% v/v PS, 20% v/v hiFBS, 1% v/v hemin, 1× BME vitamins, 4.2 mM NaHCO_3_, pH 7.2). The density of viable amastigotes that transformed and grew as promastigotes after 48 hours of culture at 26°C in 96 well microtitre plates was counted by haemocytometer. In some experiments air pouches were infected with 1×10^6^ metacyclic promastigotes for 48 hours before recovering cells. Following extensive washing to remove extracellular parasites macrophages were separated from neutrophils by adhesion to plastic for 2 hours at 37°C in DMEM medium supplemented with 20% hiFCS. Viable amastigote burdens were determined by transformation assay of amastigotes liberated from 2.5×10^5^ cells using the above protocol.

### Fluorescent growth assay

Macrophage infections were processed as for the promastigote transformation growth assays except 10% v/v alamarBlue viability dye (Invitrogen) was added to the promastigote culture medium. alamarBlue is a blue non toxic dye that when metabolised by growing cells generates a fluorescent metabolite. Fluorescence was measured (excitation wavelength = 544 nm; emission wavelength = 590 nm) 48 hours following transformation and growth in promastigote culture medium using a Gemini XPS fluorescent microplate reader (Molecular Devices) and recorded with Spectramax software.

### Nitric oxide assay

Inducible nitric oxide synthase activity was determined by measuring the amount of nitrite, the stable end product of nitric oxide metabolism using the Griess reaction. Macrophage culture supernatants were collected after 48 h, mixed with equal volumes of Griess reagent (1% sulphanilamide/0.1% *N*-(1-naphthyl) ethylenediamine dihydrochloride/2.5% H_3_PO_4_) and incubated for 10 min at room temperature. The absorbance was measured at 540 nm in a microscope plate reader (Anthos). Nitrite concentration was determined using NaNO_2_ as standard dissolved in culture medium or PBS.

### Arginase assay

Arginase activity was determined by measuring the amount of urea generated from the hydrolysis of L-arginine using the method of [27]. Briefly, 5×10^5^ BMMΦ were solubilised with 200 µl lysis solution (0.1% Triton X-100, 10 mM MnCl_2_, 25 mM Tris-HCl) and the enzyme preparation was activated by heating for 10 min at 56°C. To this 50 µl 0.5 M L-arginine (pH 9.7) was added to the preparations to allow hydrolysis of the substrate and incubated at 37°C for 15–120 min. The reaction was stopped with 400 µl of H_2_SO_4_, H_3_PO_4_, H_2_O (1∶3∶7 v/v). The urea concentration was measured at 550 nm after the addition of 20 µl α-isonitrosopropiophenone (dissolved in 100% ethanol), followed by heating at 100°C for 45 min. One unit of enzyme activity is defined as the amount of enzyme that catalyses the formation of 1 µmol of urea per min.

### Western blotting

3 µg total protein from 5×10^5^ BMMΦ released into lysis buffer (0.1% Triton X-100, 10 mM MnCl_2_, 25 mM Tris-HCl) were boiled with 1∶1 with reducing sample buffer (Invitrogen) and separated on a 4–12% SDS gradient gel (Invitrogen). Following semi-dry transfer to polyvinylidene difluoride membranes (Invitrogen) the blots were incubated in PBS-Tween blocking buffer (PBS, 0.01% v/v Tween20, 5% w/v dried milk) at 4°C overnight. Following washing in PBS-Tween the blots were incubated with either mouse 1∶200 anti-Arg1 (Santa Cruz Biotechnology), 1∶500 anti-NOS (Santa Cruz Biotechnology), 1∶1000 anti-Ym1 (Stem Cell Technologies) or 1∶2500 anti-β-actin (Sigma) mAb in blocking buffer for 4 hours at room temperature with shaking. After washing the blots were probed with an appropriate HRP-conjugated secondary antibody in blocking buffer for 1 hour at room temperature with shaking, washed again, developed with the chemiluminescent Luminol reagent (Santa Cruz Biotechnology) and exposed to x-ray film (Invitrogen).

### Flow cytometry

5×10^5^ BMMΦ were fixed and stained for the surface expression of CD206 (Serotec) according to the supplier's protocols [28]. All flow cytometry was performed on a FACSCalibur (Becton Dickinson) and analysed using Summit 4.0 software.

### Capillary feeding and PSG dot-blots

Day 8 infected sand flies were capillary force-fed 4 µl PBS for 10 min according to a previously described method [29]. Following feeding parasites were concentrated by centrifugation (3,000 rpm, 5 mins) and counted using a haemocytometer. The remaining supernatant was spotted on to positively charged nitrocellulose and processed for PSG detection. Blots were blocked overnight with TTBS (100 mm Tris-HCl, 0·9% (w/v) NaCl, 0.1% (v/v) Tween 20, pH 7.5) with 5% w/v dried milk at 4°C with shaking. Each membrane was incubated with polyclonal antisera raised against PSG in rabbit or normal rabbit serum (NRS) diluted 1∶20 in PBS-Tween for 2 hours at room temperature, washed in TTBS, and incubated with 1∶200 biotinylated anti-rabbit IgG diluted in TTBS. The membranes were labelled using ABC Elite reagent and stained with VIP substrate (Vector Laboratories Inc., UK). Images were scanned into ImageJ software (NIH) for analysis of the intensity of each PSG dot-blot.

### Statistics

Unpaired Students t-test was used to test the statistical significance between groups using the PRISM software (version 5). All comparisons were made to the saline control infections except those indicated on the graphs with a connecting line. Significance was considered as P<0.05.

## Results

### Sand fly infection intensity correlates with the amount of PSG egested and enhances low and high dose *L. mexicana* infections in mice

**Figure 1:**
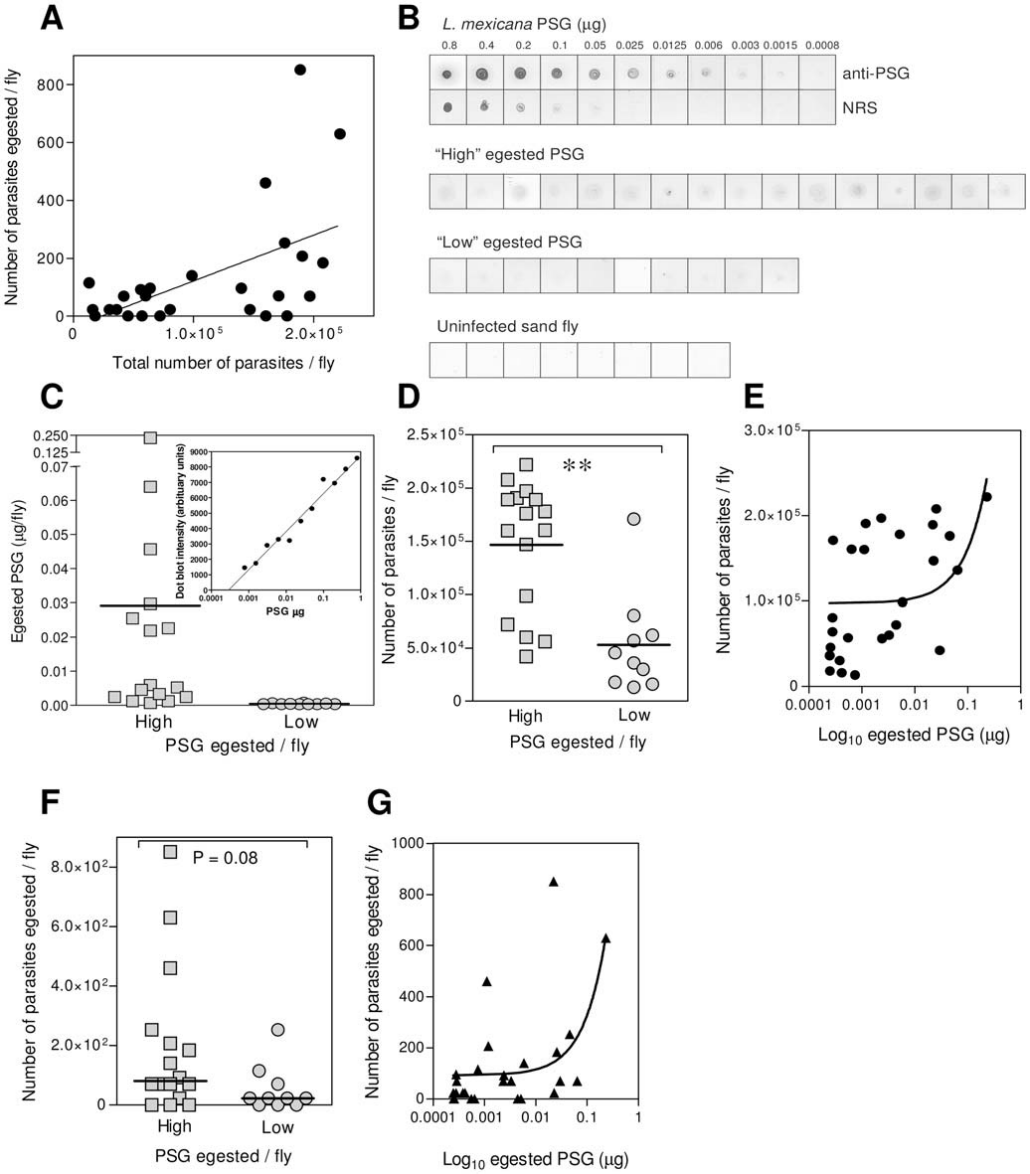
Transmitted *L. mexicana* dose in relation to amount of PSG regurgitated by sand flies. (A) Relationship between numbers of *L. mexicana* egested by *Lu. longipalpis* sand flies collected by capillary feeding and size of the sand fly midgut parasite load quantified by direct counting immediately after capillary feeding. (B) Dot-blots of *L. mexicana* PSG standards and capillary feeds from *L. mexican*a-infected sand flies probed with anti-PSG sera or normal rabbit serum (NRS). (C) Area under curve analysis of digitized PSG standards (inset) reveals high and low egestion of PSG. (D&E) Relationship between the total midgut parasite load and the amount of PSG egested. (F&G) Relationship between the transmitted dose of parasites and the amount of PSG egested. **, P<0.005.

Recently it has been shown that the dose of *L. major* parasites delivered by a sand fly relies to an extent on the size of the original sand fly infection [4]. PSG accumulates during *Leishmania* metacyclogenesis to obstruct the sand fly midgut. This blockage can greatly influence the feeding ability of the fly and limit the amount of blood intake [5],[30], forcing it to regurgitate PSG and parasites during transmission. Therefore, in accordance with these observations we hypothesised that the quantity of PSG egested correlates with the size of the sand fly infection. In order to test this we used the experimental combination of *L. mexicana* with *Lu. longipalpis*, a parasite-vector combination that is (i) fully permissive for parasite development, (ii) readily transmitted to mice by bite and (iii) generates large infections and large amounts of PSG plug [5]. To standardise the feeding process we capillary fed saline to day 8 infected sand flies for 10 minutes, and only those flies for which active feeding (cibarial pumping) was observed were included in the analysis. Following feeding the number of egested parasites was counted and each fly was dissected to determine the number of promastigotes remaining in the midgut. Using this method we observed that the number of *L. mexicana* egested positively correlated with the size of the fly infection (Fig. 1A: r^2^ = 0.28, P = 0.005). We have previously ascertained that the capillary feeding technique underestimates the dose of transmitted parasites by a factor of 10 [3]. Bearing this in mind these flies exhibited a roughly bimodal distribution such that two groups of flies with high (Average±S.E.: 1.47×10^5^±1.5×10^4^) and low (5.2×10^4^±1.48×10^4^) *Leishmania* infections were observed to transmit correspondingly large (195±240) and small (53±79) doses of parasites. By analysing the regurgitate of each fly by semi-quantitative dot-blot we could group these flies into those that regurgitated high (0.03±0.06 µg) and low (0.0004±0.00016 µg) amounts of PSG (Fig. 1B&C). This revealed that sand flies egesting high amounts of PSG had higher parasite infections (Fig. 1D: P = 0.003; Fig. 1E: r^2^ = 0.18, P = 0.0314), and importantly, these flies tended to transmit more parasites (Fig. 1F: P = 0.08; Fig. 1G: r^2^ = 0.28, P = 0.006). Previously we have shown that the quantity of PSG in a sand fly midgut is dictated by the number of leptomonad promastigotes in the infection [2],[5]. Therefore, collectively these results verify our hypothesis and show that the size of the PSG blockage determines the number of parasite transmitted and the proportion of co-regurgitated gel.

**Figure 2:**
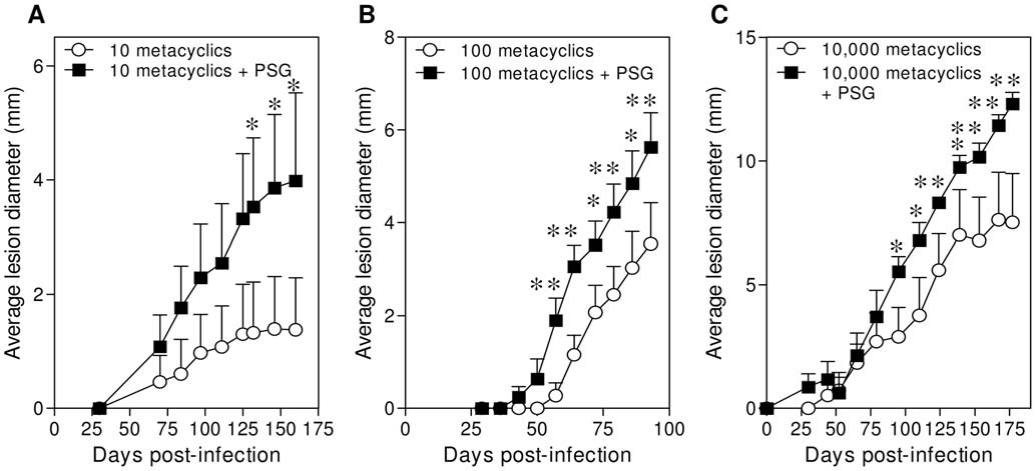
PSG exacerbates low and high dose *L. mexicana* infections in mice. (A–C) Co- inoculation of *L. mexicana* PSG (1 µg) with 10 (A), 100 (B) and 10,000 (C) *L. mexicana* metacyclic promastigotes in the rump of BALB/c mice (6 mice per group) resulted in earlier onset of lesion appearance and faster lesion evolution, displaying higher final parasite burdens (shown in Table 1). *, P<0.05; **, P<0.005.

The dose of *Leishmania* parasites delivered by infected sand flies can vary widely, such that a small proportion of sand flies can transmit either low (10–100 metacyclic promastigotes) or high-doses (5×10^3^–1×10^5^ metacyclic promastigotes) [4]. Therefore, we next examined how PSG influenced the course of *L. mexicana* infection over a range of high- and low-dose transmission scenarios. Based on our results above (taking into account that the capillary method represents only 10% of the true infective dose) we estimate that *Lu. longipalpis* could egest a maximum of 0.9 µg *L. mexicana* PSG. Figure 2 shows in BALB/c mice the evolution of dermal rump infections with 10, 100 and 1×10^4^ *L. mexicana* metacyclic promastigotes in the presence and absence of 1 µg *L. mexicana* PSG. For each dose the presence of PSG resulted in clear exacerbation of infection. In the presence of PSG lesions evolved faster, were more pronounced and harboured higher average parasite burdens at the end of the experiment (Table 1). Furthermore, the presence of PSG increased the proportion of mice that developed lesions after infection with lower doses of parasites (Table 1). These results show that the presence of regurgitated PSG, although variable, plays an important role in the establishment of infection and enhances parasite burdens after low and high dose infections.

**Table 1:** Final parasite burden in lesions of BALB/c mice inoculated with low (10, 100) or high (10,000) doses of *L. mexicana* metacyclic promastigotes with or without 1 µg *L. mexicana* PSG.

Inoculum: Dose±PSG	Lesion Parasite Burden (Average±S.E.)	Proportion of Mice Displaying Lesions
10	6.6×10^6^±2.08	3/6 (50%)
10+PSG	0.91×10^8^±2.4	4/6 (67%)
100	0.79×10^8^±0.6	4/6 (67%)
100+PSG	3.41×10^8^±0.74	6/6 (100%)
10,000	3.2×10^8^±0.52	6/6 (100%)
10,000+PSG	5.72×10^8^±0.36	6/6 (100%)

### PSG enhances the recruitment and infection of macrophages in the skin

**Figure 3:**
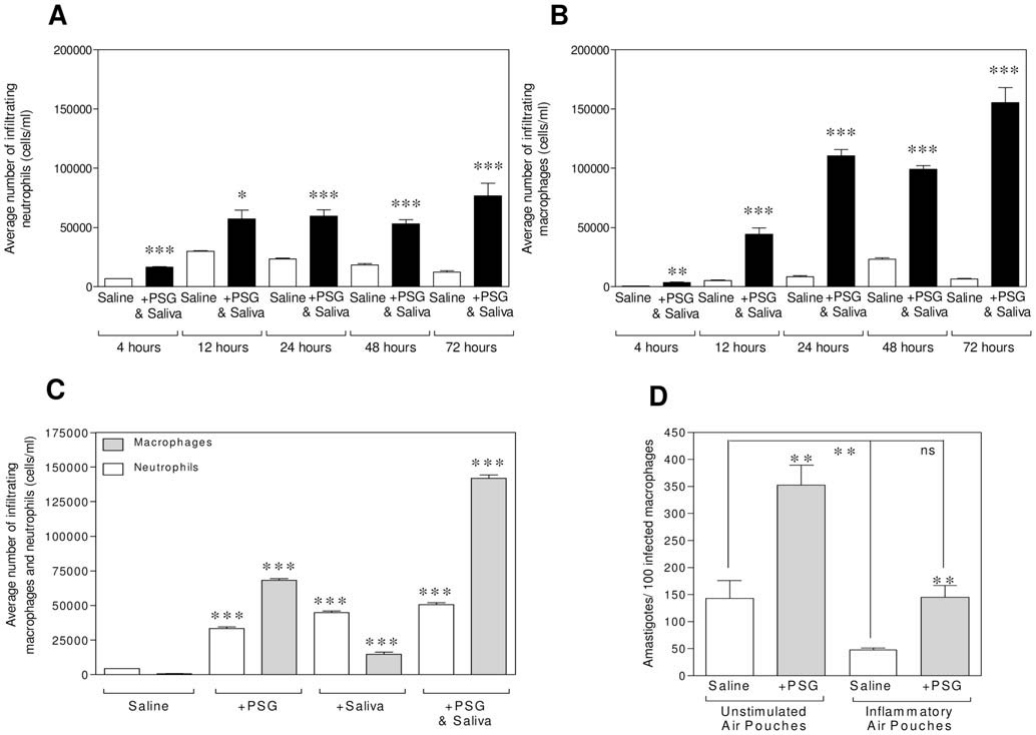
PSG recruits macrophages to the site of transmission and enhances their infection with *L. mexicana*. (A–C) Cellular content within air pouches inflated on the backs of BALB/c mice. Seventy two hour kinetic of neutrophil (A) and macrophage (B) recruitment in response to 1×10^3^ *L. mexicana* metacyclic promastigotes co-injected with 1 µg *L. mexicana* PSG and 1 µg *Lu. longipalpis* saliva. (C) Neutrophil and macrophage content of air pouches in response to 1 µg PSG, 1 µg sand fly saliva or 1 µg of PSG and saliva after 24 hours. Cell type was determined by morphology of at least 200 Giemsa-stained cells per air pouch (4–6 mice per group). Data representative of duplicate (A&B) or triplicate (C) experiments. (D) Macrophages recovered from 48 hour PSG -loaded inflammatory air pouches were infected *ex vivo* at a MOI of 1∶1 for a further 48 hours. Inflammatory air pouches were generated by the injection of 200 U INFγ and 500 U TNFα 18 hours prior to the injection of PSG. Parasite burden (D) was determined by microscopy of at least 200 Giemsa-stained macrophages in quadruplicate. Representative experiment of duplicates is shown. Unless they are linked with a bar all test groups are compared to their relevant saline control; *, P<0.05; **, P<0.005; ***, P<0.0005.

Sand fly saliva is known to attract macrophages to the site of infection [31]; however, the ability of PSG to recruit cells is unknown. To answer this question we tested the recruitment of cells to the site of PSG deposition *in vivo* using the air pouch model over a 4 to 72 hour period. Our results revealed that the combined presence of 1,000 *L. mexicana* metacyclic promastigotes, PSG and *Lu. longipalpis* saliva induced an early and significant attraction of neutrophils and macrophages to the skin compared to the injection of parasites in saline (Fig. 3A&B). In contrast, very few lymphocytes (<10%), eosinophils and basophils (<1%) were recruited to the air pouches under any of the experimental conditions used. By mimicking natural infection by sand fly bite in this way an enhanced cellular recruitment was observed as early as 4 hours post injection (neutrophils, Fig 3A: P<0.0001; macrophages, Fig. 3B: P = 0.0011) and was sustained for the remaining 72 hours. In the next step the contribution of the two main components delivered by sand fly bite to recruit cells, PSG and saliva, were determined alone or in combination. Injection of saline alone recruited mainly neutrophils to the air pouch, indicative of the needle injury to the skin. Compared to the saline group, injection of *Lu. longipalpis* saliva appeared to enhance this response by inducing a significant recruitment of both neutrophils and macrophages to the air pouch after 24 hours (Fig. 3C: neutrophils: P<0.0001; macrophages: P = 0.0007). The presence of PSG on the other hand proved to be highly efficient for attracting macrophages (P<0.0001), recruiting some 108-fold and 5-fold more macrophages to the air pouch compared to the saline control or saliva injected groups, respectively. When PSG and sand fly saliva were combined to represent the inoculum from a *Leishmania*-infected sand fly (without the parasites), this attracted the highest number of macrophages to the air pouch (224-fold more macrophages compared to saline controls, P<0.0001), indicating a synergy for macrophage chemotaxis.

Since macrophages are the definitive host cell for *Leishmania* we addressed the role of PSG during the early phase of *L. mexicana* infection *in vivo*. To do this we harvested and plated macrophages recruited to air pouches previously injected with or without PSG for 48 hours and infected them *in vitro* with a low MOI of *L. mexicana* metacyclic promastigotes (1∶1 parasite to macrophage ratio). By 48 hours post-infection those macrophages obtained *ex vivo* from PSG-injected air pouches displayed significantly higher proportions of cells infected (not shown) and higher parasite burdens than their saline-injected counterparts (Fig. 3D, P = 0.004). Similarly, infection of both macrophages and neutrophils attracted to PSG-conditioned air pouches was also enhanced *in vivo* (Fig. S1). In a parallel experiment we created an inflammatory environment in the air pouches by conditioning them for 18 hours with IFNγ and TNFα prior to injection of PSG (Fig. 3D). Saline-treated macrophages from these air pouches controlled *L. mexicana* infection by 48 hours (67% reduction in parasite burden, P = 0.1). The presence of PSG promoted parasite survival and exacerbated the 48 h infection in both unstimulated (P = 0.004) and inflammatory air pouch macrophages (P = 0.005). Moreover, PSG in inflammatory air pouches prevented the killing of *L. mexicana* in this ex vivo infection assay. Collectively, these results show that the presence of PSG (co-delivered with sand fly saliva) significantly impacts on the recruitment of host cells to the site of transmission and early *Leishmania* infection of the skin, irrespective of its inflammatory state.

### Mechanism of PSG exacerbation of infection

**Figure 4 ppat-1000555-g004:**
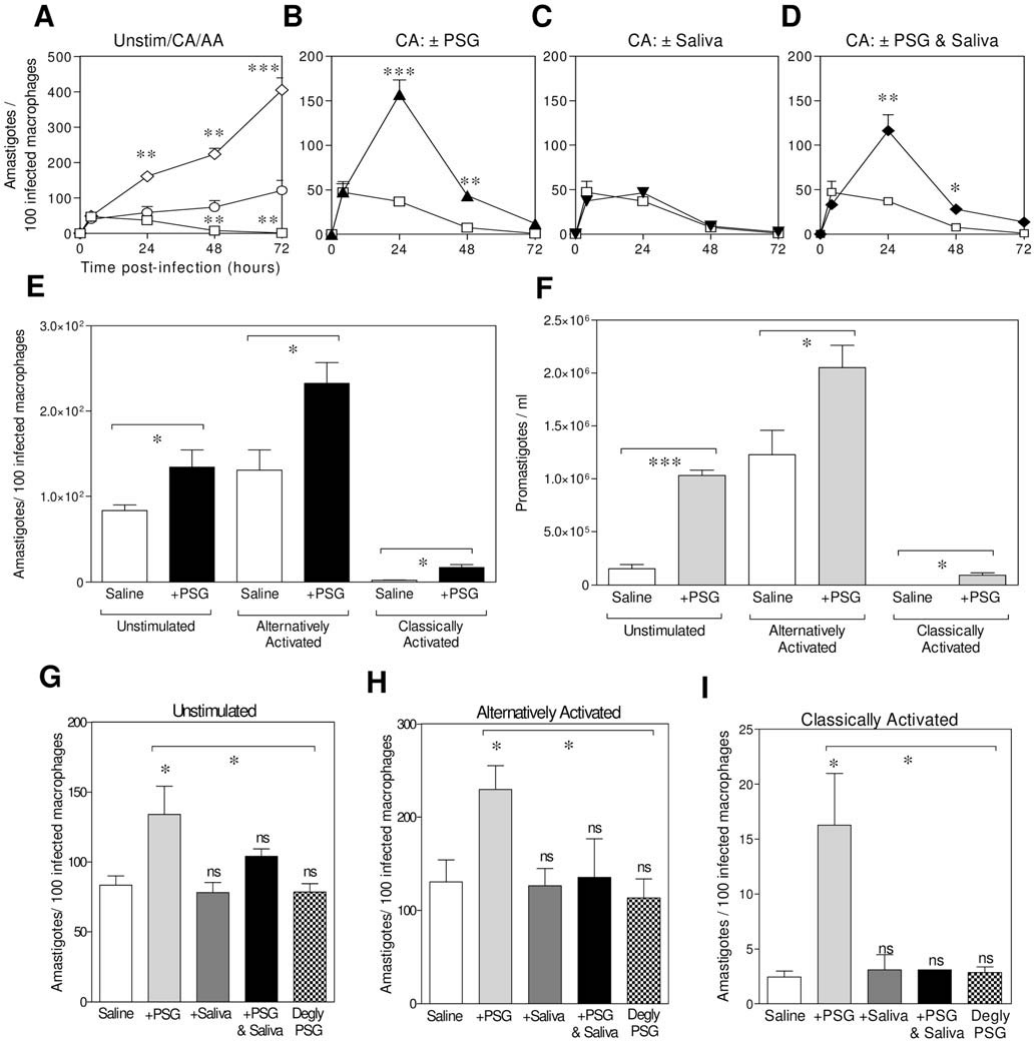
Co-incubation of *L. mexicana* with PSG enhances BALB/c macrophage infections *in vitro* under a range of activation states. (A–D) Kinetic of parasite growth in infected BMMΦ (5×10^4^; MOI 5∶1) in the presence or absence of classical activators and vector-derived products. (A) Control *L. mexicana* infections of PBS (unstimulated, open circles), IFNγ and TNFα-treated (classically activated: CAMΦ, open squares) or IL-4-treated (alternatively activated: AAMΦ, open diamonds) macrophages. The role of *L. mexicana* PSG (0.25 µg) (B), *Lu. longipalpis* sand fly saliva (0.25 µg) (C) or a combination of PSG and saliva (0.25 µg of each) (D) (closed diamonds) was assessed for infection in CAMΦ. (E&F) 48 hour parasite burden of unstimulated BMMΦ, AAMΦ and CAMΦ infected in the presence or absence of 0.25 µg PSG macrophages was assessed by microscopy of infected cells (E), or by transformation assay of amastigotes released from macrophages (F). Growth was determined by direct counting of transformed promastigotes by haemocytometer in triplicate. (G–I) Effect of PSG (0.25 µg), saliva (0.25 µg), PSG and saliva (0.25 µg of each), deglycosylated (degly) PSG (0.25 µg) or saline on LPG2^−/−^ (phosphoglycan-deficient) *L. mexicana* infections of unstimulated (G), AA (H) and CA (I) macrophages. Amastigote burden of infected macrophages determined by microscopy of at least 200 Giemsa-stained cells in triplicate or quadruplicate. Data representative of triplicate experiments. Unless they are linked with a bar all test groups are compared to their relevant saline control; ns, not significant P>0.05;*, P<0.05; **, P<0.005; ***, P<0.0005.

Knowing that PSG enhanced *L. mexicana* infections *in vivo* and that it can recruit its ultimate host cells, macrophages, we next wanted to dissect the mechanism by which PSG exacerbates *Leishmania* infections in macrophages, by modelling natural infection *in vitro* (Fig. 4). Mature BMMΦ were infected and polarized into classically or alternatively activated macrophages in the presence or absence of PSG and saliva. As expected, classical activation resulted in significant control and alternative activation exacerbated *L. mexicana* growth from 24 hours post-infection onwards, as compared to parasite growth in unstimulated macrophages (Fig. 4A). Intra-macrophage growth was assessed microscopically by counting amastigotes (Fig. 4A–E, G–I) and parasite viability was determined by transformation and growth of amastigotes released from macrophages *in vitro* (Fig. 4F), or by fluorescent growth assay (Fig. S2). Interestingly, the presence of PSG significantly increased survival in classically activated macrophages during the first 48 hours of infection (Fig. 4B, P = 0.0002). Both the proportion of macrophages infected (Fig. S3) and their parasite burdens (Fig. 4B, E & I) were increased in the presence of PSG during this time (4–8 fold increase). By contrast, *Lu. longipalpis* sand fly saliva had minimal influence, with only a moderate increase in the proportion of macrophages infected by 24 hours post infection (Fig. S3). Interestingly, the combination of PSG and saliva resulted in an intermediate pattern of infection that overall resembled the kinetics of adding PSG alone, such that infections were exacerbated for both the 24 and 48 hour time points (Fig. 4D). By 72 hours of exposure to classical activators all macrophage infections were significantly reduced and no differences were exhibited between the groups. In separate experiments, 48 hour *L. mexicana* infections revealed a clear enhancing effect of PSG on the growth and viability of *L. mexicana* infection in either unstimulated, AAMΦ or CAMΦ *in vitro* (Fig. 4E&F).

Macrophage infections with *LPG2^−/−^ L. mexicana* parasites, that are fully able to infect and grow within macrophages but unable to synthesise all phosphoglycans [25], confirmed the infection-enhancing role of the secreted proteophosphoglycans within the PSG (Fig. 4G–I). Furthermore, deglycosylation of the PSG by mild acid hydrolysis removed its ability to enhance macrophage infections with WT (not shown) and *LPG2^−/−^ L. mexicana* (Fig. 4G–I), indicating a significant role for the glycans within PSG. Collectively, these results identify a window of time *in vitro* when the presence of PSG regurgitated from the infected sand fly can promote *L. mexicana* infection in macrophages.

**Figure 5:**
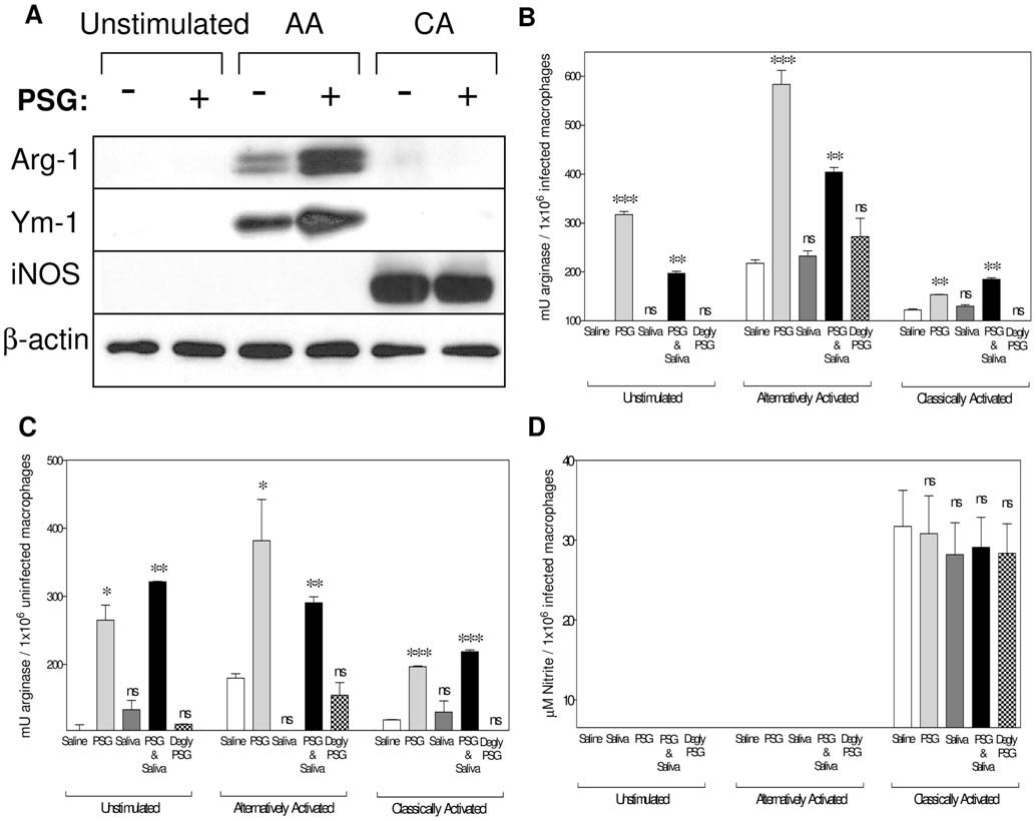
Exposure to PSG increases the alternative activation and arginase activity of macrophages. (A) 5×10^5^ unstimulated, alternatively activated and classically activated BALB/c BMMΦ were incubated in the presence or absence of 1 µg *L. mexicana* PSG for 48 hours. Western blot of macrophage lysates (3 µg protein/lane) were probed for murine Arginase-1, Ym-1, iNOS and β-actin was used as loading control. (B&C) Arginase assay of *L. mexicana*-infected (B) or uninfected (C) macrophage lysates (unstimulated, AA or CA). (D) NO assay of infected macrophage culture supernatants. Macrophages were co-incubated with 1 µg *L. mexicana* PSG, 1 µg *Lu. longipalpis* sand fly saliva or 1 µg PSG and saliva for 48 hours. Data is representative of duplicate (A) or triplicate (B–D) experiments and normalised to the average unistimulated+saline control macrophage arginase/iNOs activity. Unless they are linked with a bar all test groups are compared to their relevant saline control; ns, not significant P>0.05; *, P<0.05; **, P<0.005; ***, P<0.0005.

Neither PSG nor sand fly saliva, alone or in combination, could affect the phagocytosis of *L. mexicana* metacyclic promastigotes into macrophages (Fig. S4A&B), or the biogenesis of the macrophage phagolysosome *in vitro* (Fig. S5A–L). Instead our results from air pouch macrophages (Fig. 3C&D) suggest that PSG may condition macrophages for enhanced parasite survival soon after sand fly delivery. Western blot revealed that PSG did not dampen classical activation in macrophages, as shown by unaltered levels of iNOS expression, instead, it enhanced the alternative activation of macrophages. This was shown by an increased arginase-1 and Ym-1 expression (Fig. 5A), and increased surface expression of CD206, the murine mannose receptor (Fig. S6) in IL-4 treated cells (AAMΦ). To examine this effect further, the activity of two key macrophage enzymes involved in *Leishmania* killing or *Leishmania* proliferation: iNOS and arginase were measured in response to PSG, saliva, PSG and saliva or deglycosylated PSG. We found that the arginase levels of *L. mexicana*-infected unstimulated, AAMΦ or CAMΦ *in vitro* significantly increased in the presence of PSG (Fig. 5B, P = 0.007–0.0003). The same effect was demonstrated for uninfected macrophages indicating that the PSG can enhance the alternatively activated state before infection (Fig. 5C, P = 0.045–0.003). By contrast, saliva from the vector *Lu. longipalpis* did not influence the arginase activity of macrophages under a range of activation states tested (unstimulated, CA, AA), or *L. mexicana* infection (Fig. 5B&C). Furthermore, no synergy between PSG and saliva was observed for arginase activity except for a mild additive effect in the CAMΦ (infected and uninfected) and in unstimulated, uninfected macrophages (Fig. 5B&C). Compared to the native PSG, deglycosylated PSG could not significantly increase arginase activities of macrophages under any of the activation stated tested in the presence or absence of infection (Fig. 5B&C). In CAMΦ, neither *L. mexicana* PSG nor *Lu. longipalpis* saliva, alone or in combination, in the presence or absence of infection could affect the activity of iNOS and the generation of the leishmanicidal metabolite NO (Fig. 5D). These results indicate that PSG can enhance the capacity of their host cells to support *Leishmania* infection by modulating their arginase activity.

### PSG enhances transmissible macrophage infections via arginase in vivo

**Figure 6:**
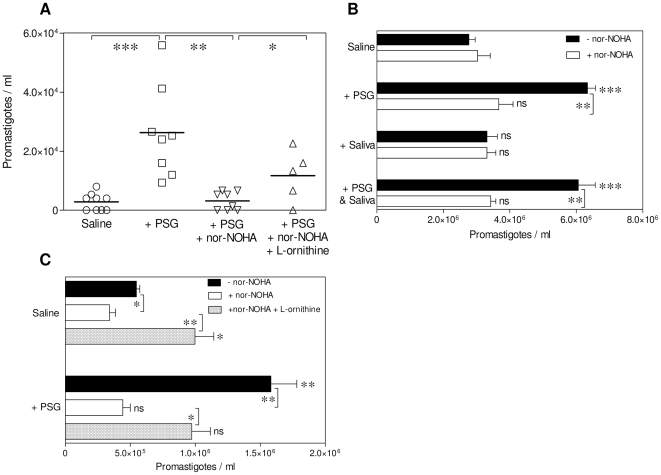
PSG promotes *L. mexicana* infection of mice by increasing the arginase activity of macrophages at the site of infection. (A) Parasite burden of BALB/c air pouches infected with 1×10^6^ *L. mexicana* metacyclic promastigotes ±1 µg *L. mexicana* PSG, ±100 µg nor-NOHA, ±100 µg L-ornithine for 48 hours. Macrophages were recovered from infected air pouches and separated by adherence to plastic. (B&C) Effect of vector-derived products on macrophage parasite burden following arginase inhibition with nor-NOHA. BALB/c BMMΦ were infected with *L. mexicana* metacyclic promastigotes at a MOI 1∶1 in the presence of ±1 µg PSG, ±1 µg *Lu. longipalpis* saliva or PSG and saliva; ±100 µM nor-NOHA; ±100 µM (B); or L-ornithine (C) for 48 hours. Viable amastigote burdens of macrophages infected *in vivo* (A), or *in vitro* (B&C) was determined by transformation assay of amastigotes liberated from either 2.5×10^5^ air pouch macrophages (4 or 8 mice per group) (A), or from 5×10^5^ *in vitro* infected BMMΦ (B&C). Data is pooled from duplicate experiments (A), or representative of duplicate experiments (B&C). Unless they are linked with a bar all test groups are compared to their relevant saline control; ns, not significant P>0.05; *, P<0.05; **, P<0.005; ***, P<0.0005.

The results presented in Figures 3–5 indicate that PSG promotes the early survival and growth of *L. mexicana* parasites *in vivo* by enhancing the arginase activity of macrophages recruited to the site of infection. To demonstrate that higher arginase activities of macrophages exposed to PSG translated into exacerbated infections *in vivo* we infected air pouches with 10^6^ *L. mexicana* metacyclic promastigotes on BALB/c mice treated or not treated with the selective competitive inhibitor of arginase, nor-NOHA. Transformation and growth of amastigotes (as promastigotes) released from air pouch macrophages revealed that those mice that received PSG without nor-NOHA treatment displayed the highest viable parasite burden (Fig. 6A, P = 0.005), confirming the exacerbatory role of PSG *in vivo*. Inhibition of arginase activity by nor-NOHA significantly inhibited the exacerbating effect of PSG in these mice by 87%, demonstrating that the ability of PSG to increase macrophage arginase levels is key to it's disease enhancing properties *in vivo* (P = 0.0011). The specificity of nor-NOHA for mammalian and not parasite arginase was tested by culturing *L. mexicana* axenic amastigotes and promastigotes in the presence of nor-NOHA (Fig. S7A&B and Table S1). Arginase controls the production of polyamines within macrophages via the metabolism of L-arginine, and these are essential nutrients for intracellular amastigote growth [11]. The first step along this pathway is the hydrolysis of L-arginine to L-ornithine, catalysed by arginase. Supplementation of air pouches with L-ornithine could bypass the requirement for L-arginine and arginase and enhanced the growth of *Leishmania* in the presence of nor-NOHA (P = 0.025), indicating that PSG exerted its effects via arginase activity and subsequent synthesis of polyamines *in vivo*. Next, we repeated these experiments with cultured macrophages and assessed the effects of PSG on amastigote viability (Fig. 6B&C). Viability was determined by the transformation of amastigotes to flagellated promastigotes *in vitro*, a process that mimics the next stage in the *Leishmania* life-cycle in the sand fly vector. We found that amastigotes liberated from unstimulated BMMΦ exposed to PSG, either alone or in combination with *Lu. longipalpis* saliva, were more viable on a per parasite basis compared to those exposed to saline (Fig. 6B&C, P = 0.0021−<0.0001). By comparison, amastigotes from macrophages exposed to saliva on its own did not display any change in their viability as compared to saline controls (Fig. 6B). Co-incubation of macrophages with nor-NOHA reduced the benefit of PSG exposure to amastigote growth by 58–72% (P = 0.0015), and could be partially rescued by supplementation with L-ornithine (Fig. 6C). Whatever their origin - air pouch cavity-derived MΦ or BMMΦ- PSG-exposed MΦ displayed nor-NOHA sensitive arginase activity.

## Discussion

The findings of the present report reveal a crucial role for PSG delivered by infected sand flies on macrophages and the early establishment of leishmaniasis in the skin. We identify two key mechanisms behind this exacerbation and reveal that PSG recruits macrophages to the site of infection and increases their expression of arginase.

To begin to understand the properties of gel in the initiation of infection we first addressed the dynamics of PSG regurgitation from individual sand flies. Recently it has been shown using real time quantitative RT-PCR of bitten skin that *P. papatasi* sand flies infected with *L. major* parasites can deliver a wide range of parasites, from 100 to 100,000, and that high dose infections were associated with heavy midgut infections of greater than 30,000 parasites [4]. We confirm this finding using the experimental, yet permissive and transmissible sand fly-parasite combination of *Lu. longipalpis* and *L. mexicana*. By using a capillary feeding technique we also show that the number of parasites inoculated corresponds to the size of the original midgut infection. Although the capillary feeding technique does not replicate many aspects of feeding on skin and we have shown that it is some 10-fold less sensitive than measuring the dose of infecting parasites compared to membrane feeding [3], it does however allow us to standardize the duration of feeding and to collect egested PSG on an individual sand fly basis. Taking into account the underestimation of parasite and PSG dose with this method [3] we show they were closely associated with the sand fly infection intensity. In accordance with Kimblin *et al* [4] the distribution of parasite doses was roughly bimodal, such that sand flies appeared to transmit large doses of parasites when the original midgut infection intensity was over ∼1.5×10^5^ promastigotes per fly. This fits with “blocked-fly” hypothesis of *Leishmania* transmission, which identifies a mode of transmission other than inoculation of parasites from within the sand fly foregut and proboscis [3],[5],[7]. Rather, higher numbers of parasites are regurgitated from the midgut, embedded within the PSG plug. These results also highlight the possibility that sand flies can egest differing doses of PSG and parasites which may influence the course of infection. This seems likely because we know that blockage of the sand fly midgut with PSG can radically alter the feeding behaviour of the sand fly such that it takes longer to feed and is more persistent in its feeding [5],[32]. These changes would in turn favour the deposition of more parasites, PSG and possibly sand fly saliva. From this study we found that high and low dose transmitting flies egested a correspondingly high and low amount of PSG, indicating that the size of the sand fly infection, dose of transmitted parasites and dose of regurgitated PSG are linked. The influence of the dose of infecting parasites on the course of infection was also highlighted by Kimblin *et al* [4]; however, the role of PSG in this process is unknown. Here we show that PSG can significantly exacerbate lesion development and parasite growth *in vivo* over a wide range of possible infectious doses (10–10^4^ *L. mexicana* metacyclic promastigotes). Using experiments of this kind it will be possible to tailor the parasite dose (using sand fly derived parasites to ensure optimal infectivity) with physiologically relevant amounts of PSG and saliva to model their role in natural infection more accurately. Collectively these results highlight the important role that the PSG blockage plays in influencing the parasite dose and facilitating cutaneous infection.

To identify how PSG enhances cutaneous leishmaniasis we focused on the first few days of infection since this is the time when the parasite are most vulnerable to potent innate immune responses to the sand fly bite and when PSG, saliva and parasites are present together in the skin. Firstly, we found that PSG was able to strongly influence macrophage recruitment. This effect was rapid and was observable by 4 hours. Sand fly bite and sand fly saliva has also been shown to recruit both neutrophils and macrophages in the skin [16],[31]. We show that the combination of PSG and saliva was additive and recruited even higher numbers of cells to the site of injection, with a bias still in favour of macrophages. Both neutrophils and macrophages can host *Leishmania* [16],[33] and in this way the sand fly bite, saliva and parasite PSG act synergistically to provide more potential host cells to the site of infection.

Secondly, PSG proved to greatly influence the early course of infection in macrophages both *in vitro* and *ex vivo* as well as in macrophages *in situ* within the air pouch. We found that the gel rapidly conditioned macrophages for enhanced parasitism in advance of infection and even reduced the efficiency of parasite elimination in CAMΦ. This latter finding is especially relevant to leishmaniasis since the catabolism of L-arginine by iNOS and the generation of NO is one of the most potent killing mechanisms of intracellular *Leishmania* parasites [23]. iNOS is induced *in vivo* in the early phase of infection [34] when macrophages respond to innate recognition pathways and the activation of toll like receptors (TLRs) [35]. It has been shown that iNOS expression can be suppressed *in vitro* by the saliva of sand flies, including *Lu. longipalpis* [36]. In our hands *Lu. longipalpis* saliva did not influence NO generation or the course of *L. mexicana* infection *in vitro*. Furthermore, PSG, either alone or in combination with sand fly saliva, did not influence NO production. Instead we found that PSG enhanced alternative activation of macrophages, increased arginase-1 expression, arginase activity and thereby ensured increased synthesis of nutrients. Two isoforms of arginase exist, arginase-1 and 2 of which the inducible, cytosolic form, arginase-1, predominates in macrophages; therefore, it cannot be completely excluded that PSG also interacts with arginase-2. However, this seems unlikely since its role in the intracellular growth of *Leishmania* in macrophages has been excluded [11]. Arginase-1 in macrophages is induced by Th2 type cytokines such as IL-4, IL-10, IL-13 and IL-21 and increased arginase is associated with resistance to worm infections, allergic conditions such as asthma and Th2 induced pathologies [37]–[40]. The intracellular growth of *Leishmania* is intimately linked with host arginase since it hydrolyses arginine into ornithine, the precursor for polyamines. In turn, polyamines are necessary nutrients for *Leishmania* and chronically high arginase activity is associated with uncontrolled parasite growth and cutaneous pathology in mice [11],[41]. This explains the reduced efficiency of macrophages from air pouches exposed to inducers of the iNOS pathway to kill *L. mexicana* in the presence of PSG and supports the finding that intracellular pathogens can survive in CAMΦ by increasing their arginase expression [42]. Addition of the competitive arginase inhibitor nor-NOHA *in vi*vo and *in vitro* demonstrated that the activity of arginase is critical to the infection enhancing properties of PSG. Furthermore, by adding L-ornithine to nor-NOHA treated cells or mice we could bypass the arginase blockade. This confirmed that it was the parasite's requirement for L-ornithine-derived polyamines that benefited the growth and survival of *L. mexicana* in macrophages *in vitro* and *in vivo*.

Injection of *P. papatasi* salivary gland sonicates can induce a rapid expression of IL-4 from mouse epidermal cells and *Lu. longipalpis* saliva can induce IL-10 expression from mouse ear tissue and cultured macrophages [43]–[45]. Both these cytokines synergize in the alternative activation of macrophages, the expression of arginase in macrophages and the promotion of parasite growth [11]. Despite this, saliva and PSG did not act additively on macrophage arginase activity or parasitism, reflecting previous infections in mice [3]. Recently, it has been shown that cytokine-dependent, STAT 6-dependent pathways as well as TLR-dependent, STAT-6 independent pathways can induce arginase-1 in macrophages depending on the type of infection [42]. Indeed, we have previously shown that increased *Leishmania* survival in TLR4-deficient host cells correlates with enhanced arginase activity [35]. Furthermore, the disparity in the cytokine responses invoked by the injection of salivary gland sonicate with *Leishmania* [17] and infected sand fly bite [19] may be due to the unaccounted presence of PSG in the latter; suggesting that PSG and sand fly saliva may indeed act via different pathways or cytokines. Therefore, it will be an important step to identify the precise signaling mechanisms involved in this parasite-vector-host interaction to understand how *Leishmania* establish infection following sand fly bite.

Arginine metabolism is critical to effective wound healing in the skin which is co-ordinated by iNOS and arginase expression [46]. During the initial inflammatory phase which normally lasts 1–2 days CAMΦ express iNOS and generate NO to control the growth of introduced pathogens. These are replaced by the infiltration of AAMΦ and myeloid cells which express arginase and promote wound repair and closure by catabolising L-arginine into L-ornithine and L-proline which in turn promote the proliferation of fibroblasts and the deposition of collagen [47],[48]. Sand flies belong to a large family of vectors (including black flies, tsetse flies, stable flies, tabanids and biting midges) known collectively as pool feeders, which obtain blood by lacerating the surface capillaries of the upper dermis [49],[50]. This causes strong inflammation and sets in motion the wound response [33]. It has been shown that arginase expression and alternative activation was found to be an innate and rapid response to tissue injury that takes place even in the absence of an infectious agent [51]. Therefore, *Leishmania*, through the regurgitation of PSG, appear to exploit this normal aspect of the wound healing for its own survival and proliferation. Since arginase and iNOS share L-arginine as substrate, enhanced arginase activity will promote parasite proliferation, modulate substrate availability for iNOS as well as its stability and reduce parasite killing [52]. Thus, the presence of PSG in the infectious inoculum helps *Leishmania* to escape early destruction in macrophages and provides a favourable environment for parasite survival and multiplication by increasing the pool of nutrients required for their growth.

To our knowledge the only study which has investigated the relationship between wound healing and leishmaniasis reported that resistance of mice to *L. major* infection was associated with high collagen deposition and a faster rate of wound healing, controlled in part by three leishmaniasis susceptibility loci mapped to chromosome 17 [53]. However, it has also been established experimentally in mice that *Leishmania amazonensis* can readily metastasize to a fresh skin cut [54] and clinical cases of new cutaneous *Leishmania* lesions following local trauma to the skin has been recorded ([55] and references therein), therefore, our understanding of leishmaniasis and the wound response is less than complete. Our results strongly suggest that PSG can modulate the early wound healing process in favor of parasite survival and growth following transmission by sand fly bite. Wound healing, however, is a highly complex and dynamic process involving many cell types, so for the future it would be important to dissect further the influence of PSG on the wound healing process itself.

An important cell type in the immediate response to dermal injury is the neutrophil. Real-time intravital imaging of sand fly transmission has recently revealed a significant role for neutrophils, rapidly attracted to the sand fly bite, for the early survival of parasites in the skin [16]. Neutrophils have been ascribed as temporary host cells for *Leishmania* which allow silent entry into macrophages when they readily ingest apoptotic infected neutrophils; the “Trojan Horse” theory of infection [56]. As our data suggest (Fig. S1) PSG can promote neutrophil infection and may participate in this interaction, which warrants further investigation. As shown in this study, and previously [3], the presence of PSG in the inocula exacerbates long-term *Leishmania* growth and lesion development in mice. The early ‘boost’ to parasite growth by PSG may therefore carry on throughout the course of infection and contribute to the persistence of parasites within the skin.

In summary, we have identified an important early survival strategy for *Leishmania* following natural transmission by sand flies. We show that the parasites can manipulate its sand fly vector to regurgitate PSG which in turn manipulates the mammalian host by the alternatively activating macrophages and increasing their arginase-mediated L-arginine catabolism. This amplification of the wound repair response to the tissue injury caused by the bite of the sand fly resulted in increased parasite survival and multiplication during the initial phase of infection. Based on the data presented here we hypothesize that PSG could play an important role in the infection of saliva-immune individuals living in endemic areas.

## Supporting Information

Figure S1 PSG enhances the viability of *L. mexicana* inside air pouch macrophages and neutrophils *in vivo*. Cells recruited to BALB/c air pouches inoculated with 1×10^6^ *L. mexicana* metacyclic promastigotes with (closed symbols) or without (open symbols) 1 µg *L. mexicana* PSG for 48 hours were recovered, washed and plated on plastic to harvest adherent macrophages (circles) and non-adherent neutrophils (squares). Viable parasite burdens were determined by transformation assay of amastigotes liberated from 2.5×10^5^ cells per air pouch. *, P<0.05; **, P<0.005.
(0.05 MB TIF)

Figure S2 Co-incubation with PSG enhances the viability of *L. mexicana* inside macrophages *in vitro*. The viability of parasites obtained from 5×10^4^ unstimulated, AA and CA macrophages, in the presence or absence of 0.25 µg *L. mexicana* PSG was assessed 48 hour after infection by labeling with fluorescence. Amastigotes released from macrophages were transformed and grown as promastigotes in the presence of 10% alamarBlue fluorescent viability dye for 48–72 hours. Infected macrophages were assayed in triplicate or quadruplicate. Data representative of triplicate experiments. ns, not significant P>0.05; *, P<0.05; **, P<0.005.
(0.06 MB TIF)

Figure S3 Co-incubation with PSG enhances the proportion of *L. mexicana*-hosting macrophages *in vitro*, under a range of activation states. (A–D) Kinetic of infected BALB/c bone marrow-derived macrophages in the presence or absence of classical activators and vector-derived products. (A) Control *L. mexicana* infections of PBS (unstimulated, open circles), IFNγ and TNFα-treated (classically activated: CAMΦ, open squares) or IL-4-treated (alternatively activated: AAMΦ, open diamonds) macrophages. The role of 0.25 µg *L. mexicana* PSG (B), 0.25 µg *Lu. longipalpis* sand fly saliva (C) or 0.25 µg of PSG and saliva (D) was assessed in infected CAMΦ (closed symbols) compared to PBS treated controls (open squares). Proportion of infected macrophages was determined by counting at least 200 Giemsa-stained cells in triplicate or quadruplicate. Data representative of triplicate experiments. *, P<0.05; **, P<0.005.
(0.05 MB TIF)

Figure S4 PSG does not influence the phagocytosis of *L. mexicana in vitro*. (A&B) Unstimulated, alternatively activated (AA) and classically activated (CA) BALB/c BMMΦ were infected with *L. mexicana* metacyclic promastigotes at MOI 5∶1 in the presence or absence of 1 µg/ml *L. mexicana* PSG, 1 µg/ml *Lu. longipalpis* saliva, 1 µg/ml PSG and saliva or PBS control for 4 hours. (A) Proportion and (B) parasite burden of infected macrophages were determined by microscopy. Proportion and amastigote burden of infected macrophages determined by microscopy of at least 200 Giemsa-stained cells in triplicate or quadruplicate. Data representative of triplicate experiments. ns, not significant P>0.05; *, P<0.05.
(0.07 MB TIF)

Figure S5 PSG does not influence macrophage phagosome-lysosome fusion during *L. mexicana* infection *in vitro*. (A–L) Confocal microscopy of phagosome-lysosome fusion after 4 hours infection with red fluorescent *L. mexicana* parasites (*L. mexicana* dsRed, MOI 5∶1) in unstimulated BMMΦ (A–D), CAMΦ (E–H) and AAMΦ (I–L). Late endosomes are labelled blue and lysosomes are labelled green. Images are in pairs: phase contrast images show position of red fluorescent *L. mexicana* inside macrophages, dark field images show position of fluorescent markers in macrophages. Right-hand panels (A+B, E+F, I+J) are PBS control infections and left-hand panels (C+D, G+H, K+L) are infections in the presence of 1 µg/ml *L. mexicana* PSG.
(0.63 MB TIF)

Figure S6 PSG increases the alternative activation of macrophages. 5×10^5^ unstimulated, alternatively activated BALB/c macrophages were incubated in the presence or absence of 0.25 µg *L. mexicana* PSG for 48 hours. Surface expression of murine mannose receptor, CD206 was determined by flow cytometry using a FACSCalibur (Becton Dickinson). Representative data is shown.
(0.06 MB TIF)

Figure S7 The arginase inhibitor nor-NOHA does not affect *L. mexicana* amastigote and promastigote growth and development *in vitro*. Lesion amastigotes were seeded into culture at 1×10^6^/ml and 5×10^5^/ml for axenic amastigote and promastigote cultures, respectively. (A and B) Growth kinetics of amastigotes (A) or promastigotes (B) grown in culture media with (closed symbols), or without (open symbols) 100 µM nor-NOHA. For each growth condition the data is pooled from 3 cultures. **, P<0.005; ***, P<0.0005.
(0.05 MB TIF)

Table S1 Key features of *L. mexicana* amastigote and promastigote growth and development *in vitro* are unaffected by the presence of the arginase inhibitor nor-NOHA. Promastigote and amastigote growth was observed for 8 days and the mean population doubling time (PDT) (fast parasite growth is reflected by a low PDT) was determined from 4 cultures per condition. The average proportion of metacyclic promastigotes was determined from Giemsa-stained smears of day 8 stationary phase promastigote cultures and the ability of stationary phase amastigotes to transform to promastigotes was similarly determined by growing them in promastigote medium for 24 hours.
(0.06 MB TIF)
