# Supplementary figures and images for: Proteophosophoglycans Regurgitated by Leishmania-Infected Sand Flies Target the L-Arginine Metabolism of Host Macrophages to Promote Parasite Survival
Source: PLoS Pathog. 2009 Aug 21;5(8):e1000555. doi: 10.1371/journal.ppat.1000555 (PMC2722086; doi:10.1371/journal.ppat.1000555)

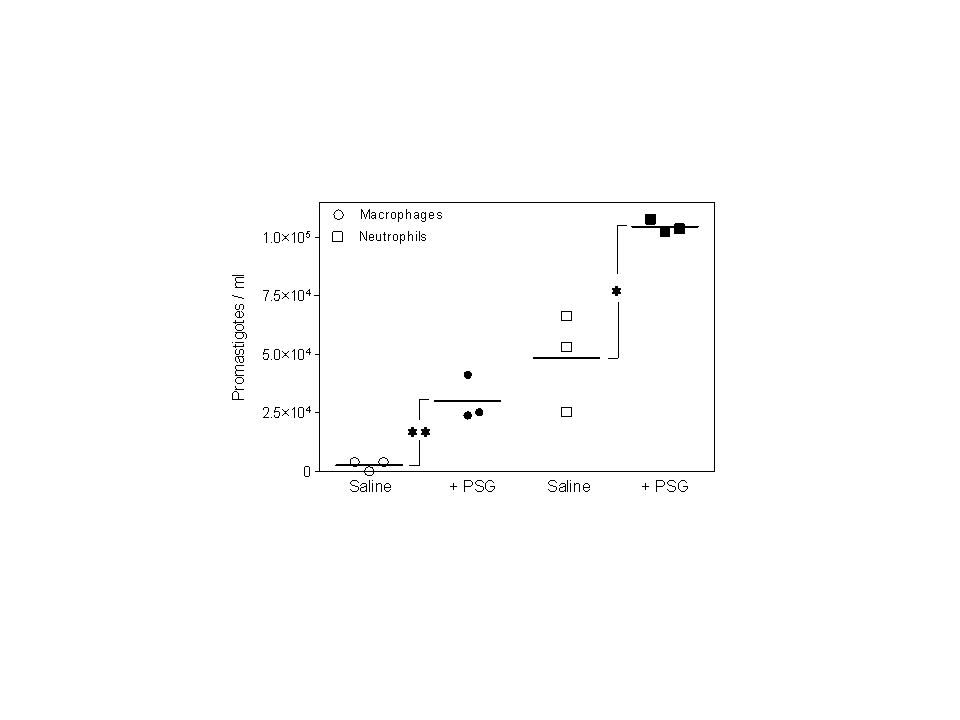

Supplement: Figure S1 — PSG enhances the viability of L. mexicana inside air pouch macrophages and neutrophils in vivo. Cells recruited to BALB/c air pouches inoculated with 1×10^6^ L. mexicana metacyclic promastigotes with (closed symbols) or without (open symbols) 1 µg L. mexicana PSG for 48 hours were recovered, washed and plated on plastic to harvest adherent macrophages (circles) and non-adherent neutrophils (squares). Viable parasite burdens were determined by transformation assay of amastigotes liberated from 2.5×10^5^ cells per air pouch. *, P<0.05; **, P<0.005. (0.05 MB TIF) [file ppat.1000555.s001.tif]

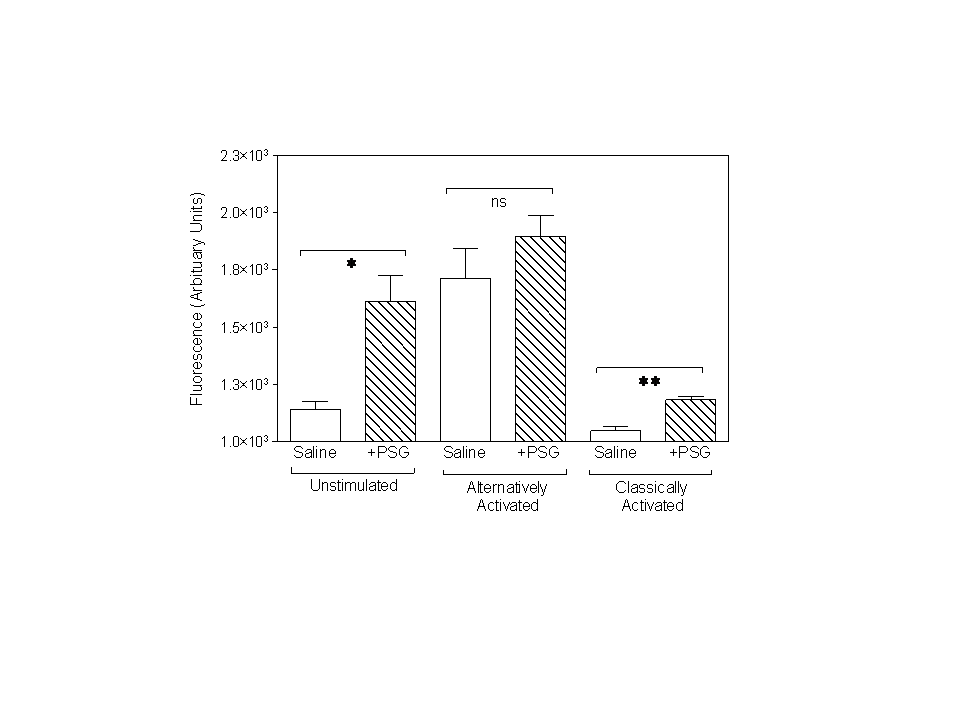

Supplement: Figure S2 — Co-incubation with PSG enhances the viability of L. mexicana inside macrophages in vitro. The viability of parasites obtained from 5×10^4^ unstimulated, AA and CA macrophages, in the presence or absence of 0.25 µg L. mexicana PSG was assessed 48 hour after infection by labeling with fluorescence. Amastigotes released from macrophages were transformed and grown as promastigotes in the presence of 10% alamarBlue fluorescent viability dye for 48–72 hours. Infected macrophages were assayed in triplicate or quadruplicate. Data representative of triplicate experiments. ns, not significant P>0.05; *, P<0.05; **, P<0.005. (0.06 MB TIF) [file ppat.1000555.s002.tif]

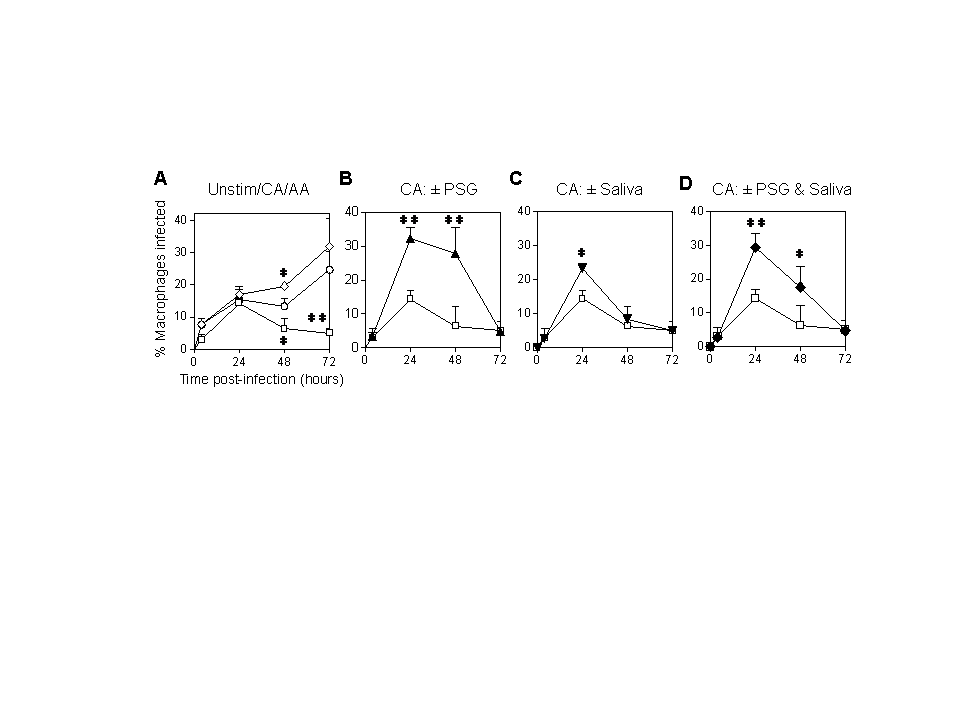

Supplement: Figure S3 — Co-incubation with PSG enhances the proportion of L. mexicana-hosting macrophages in vitro, under a range of activation states. (A–D) Kinetic of infected BALB/c bone marrow-derived macrophages in the presence or absence of classical activators and vector-derived products. (A) Control L. mexicana infections of PBS (unstimulated, open circles), IFNγ and TNFα-treated (classically activated: CAMΦ, open squares) or IL-4-treated (alternatively activated: AAMΦ, open diamonds) macrophages. The role of 0.25 µg L. mexicana PSG (B), 0.25 µg Lu. longipalpis sand fly saliva (C) or 0.25 µg of PSG and saliva (D) was assessed in infected CAMΦ (closed symbols) compared to PBS treated controls (open squares). Proportion of infected macrophages was determined by counting at least 200 Giemsa-stained cells in triplicate or quadruplicate. Data representative of triplicate experiments. *, P<0.05; **, P<0.005. (0.05 MB TIF) [file ppat.1000555.s003.tif]

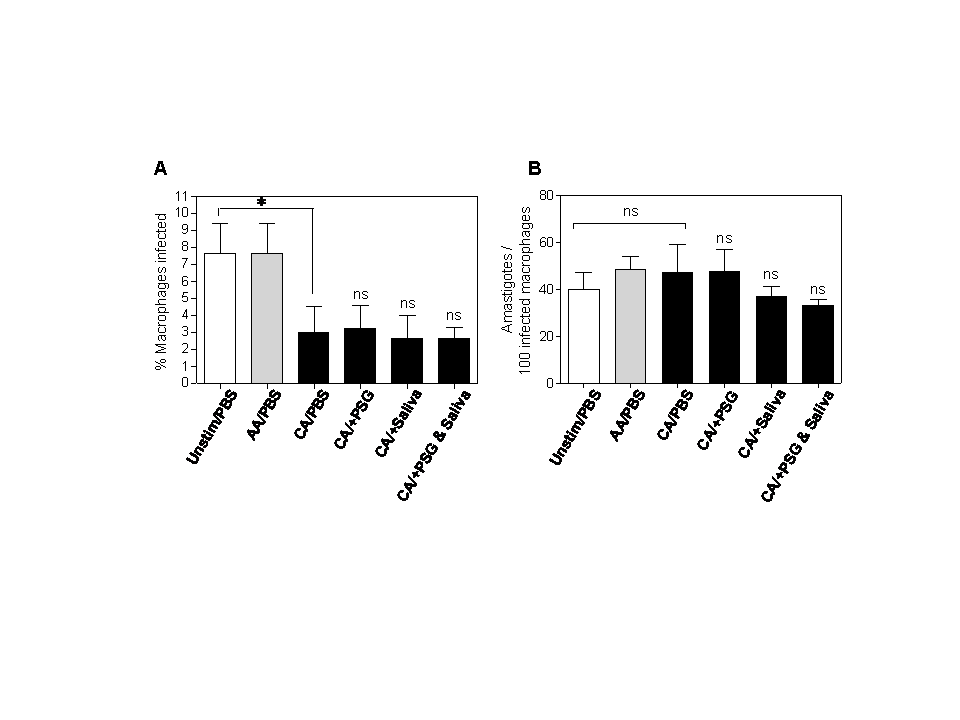

Supplement: Figure S4 — PSG does not influence the phagocytosis of L. mexicana in vitro. (A&B) Unstimulated, alternatively activated (AA) and classically activated (CA) BALB/c BMMΦ were infected with L. mexicana metacyclic promastigotes at MOI 5∶1 in the presence or absence of 1 µg/ml L. mexicana PSG, 1 µg/ml Lu. longipalpis saliva, 1 µg/ml PSG and saliva or PBS control for 4 hours. (A) Proportion and (B) parasite burden of infected macrophages were determined by microscopy. Proportion and amastigote burden of infected macrophages determined by microscopy of at least 200 Giemsa-stained cells in triplicate or quadruplicate. Data representative of triplicate experiments. ns, not significant P>0.05; *, P<0.05. (0.07 MB TIF) [file ppat.1000555.s004.tif]

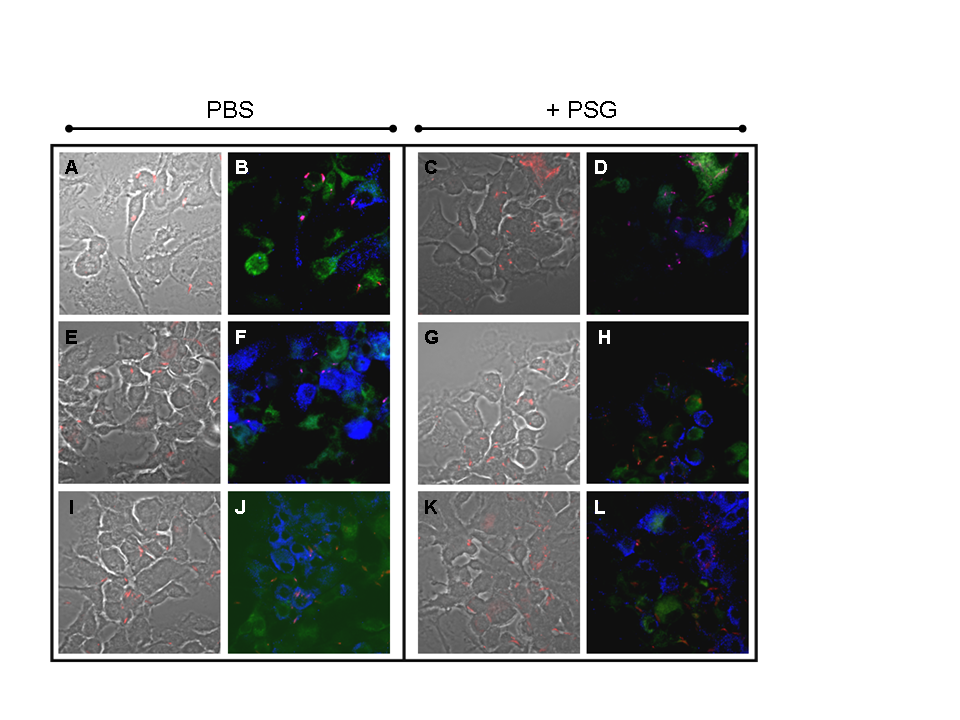

Supplement: Figure S5 — PSG does not influence macrophage phagosome-lysosome fusion during L. mexicana infection in vitro. (A–L) Confocal microscopy of phagosome-lysosome fusion after 4 hours infection with red fluorescent L. mexicana parasites (L. mexicana dsRed, MOI 5∶1) in unstimulated BMMΦ (A–D), CAMΦ (E–H) and AAMΦ (I–L). Late endosomes are labelled blue and lysosomes are labelled green. Images are in pairs: phase contrast images show position of red fluorescent L. mexicana inside macrophages, dark field images show position of fluorescent markers in macrophages. Right-hand panels (A+B, E+F, I+J) are PBS control infections and left-hand panels (C+D, G+H, K+L) are infections in the presence of 1 µg/ml L. mexicana PSG. (0.63 MB TIF) [file ppat.1000555.s005.tif]

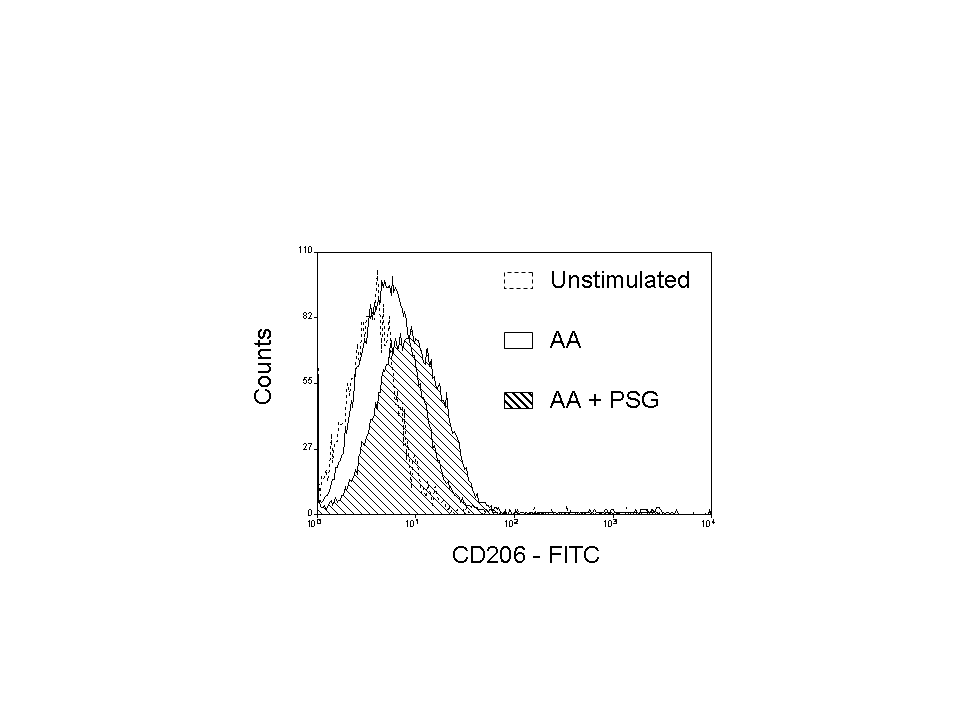

Supplement: Figure S6 — PSG increases the alternative activation of macrophages. 5×10^5^ unstimulated, alternatively activated BALB/c macrophages were incubated in the presence or absence of 0.25 µg L. mexicana PSG for 48 hours. Surface expression of murine mannose receptor, CD206 was determined by flow cytometry using a FACSCalibur (Becton Dickinson). Representative data is shown. (0.06 MB TIF) [file ppat.1000555.s006.tif]

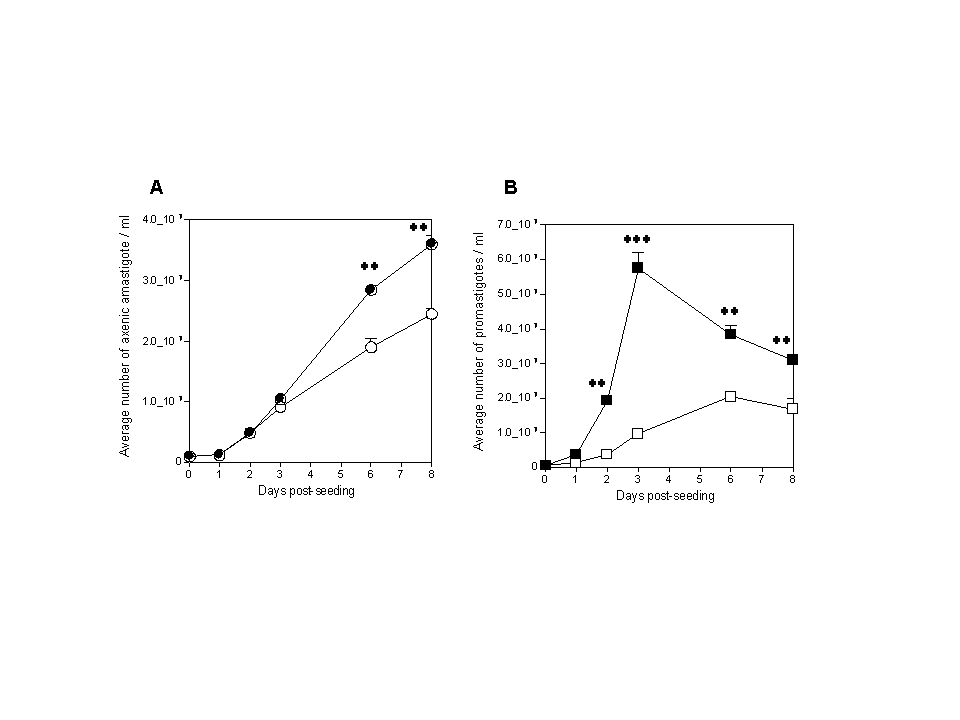

Supplement: Figure S7 — The arginase inhibitor nor-NOHA does not affect L. mexicana amastigote and promastigote growth and development in vitro. Lesion amastigotes were seeded into culture at 1×10^6^/ml and 5×10^5^/ml for axenic amastigote and promastigote cultures, respectively. (A and B) Growth kinetics of amastigotes (A) or promastigotes (B) grown in culture media with (closed symbols), or without (open symbols) 100 µM nor-NOHA. For each growth condition the data is pooled from 3 cultures. **, P<0.005; ***, P<0.0005. (0.05 MB TIF) [file ppat.1000555.s007.tif]

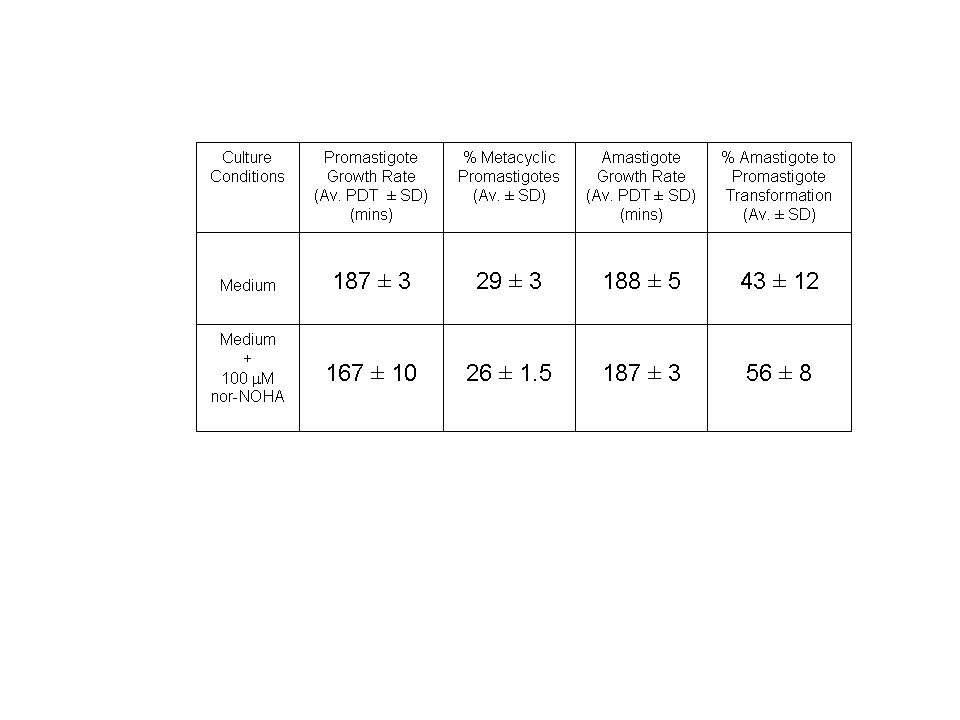

Supplement: Table S1 — Key features of L. mexicana amastigote and promastigote growth and development in vitro are unaffected by the presence of the arginase inhibitor nor-NOHA. Promastigote and amastigote growth was observed for 8 days and the mean population doubling time (PDT) (fast parasite growth is reflected by a low PDT) was determined from 4 cultures per condition. The average proportion of metacyclic promastigotes was determined from Giemsa-stained smears of day 8 stationary phase promastigote cultures and the ability of stationary phase amastigotes to transform to promastigotes was similarly determined by growing them in promastigote medium for 24 hours. (0.06 MB TIF) [file ppat.1000555.s008.tif]
